# Fluorescence resonance energy transfer (FRET)-based subcellular visualization of pathogen-induced host receptor signaling

**DOI:** 10.1186/1741-7007-7-81

**Published:** 2009-11-25

**Authors:** Alexander Buntru, Timo Zimmermann, Christof R Hauck

**Affiliations:** 1 Lehrstuhl für Zellbiologie, Universität Konstanz, Konstanz, Germany; 2 Konstanz Research School Chemical Biology, Universität Konstanz, Konstanz, Germany; 3 Advanced Light Microscopy Unit, CRG-Centre de Regulació Genòmica, Barcelona, Spain

## Abstract

**Background:**

Bacteria-triggered signaling events in infected host cells are key elements in shaping the host response to pathogens. Within the eukaryotic cell, signaling complexes are spatially organized. However, the investigation of protein-protein interactions triggered by bacterial infection in the cellular context is technically challenging. Here, we provide a methodological approach to exploit fluorescence resonance energy transfer (FRET) to visualize pathogen-initiated signaling events in human cells.

**Results:**

Live-cell microscopy revealed the transient recruitment of the Src family tyrosine kinase Hck upon bacterial engagement of the receptor carcinoembryonic antigen-related cell adhesion molecule 3 (CEACAM3). In cells expressing a CEACAM3 variant lacking the cytoplasmic domain, the Src homology 2 (SH2) domain of Hck (Hck-SH2) was not recruited, even though bacteria still bound to the receptor. FRET measurements on the basis of whole cell lysates revealed intimate binding between Hck-SH2 (using enhanced yellow fluorescent protein (YPet)-Hck-SH2) and the tyrosine-phosphorylated enhanced cyan fluorescent protein-labeled cytoplasmic domain of wild-type CEACAM3 (CEACAM3 WT-CyPet) and a flow cytometry-based FRET approach verified this association in intact cells. Using confocal microscopy and acceptor photobleaching, FRET between Hck-SH2 and CEACAM3 was localized to the sites of bacteria-host cell contact.

**Conclusion:**

These data demonstrate not only the intimate binding of the SH2 domain of Hck to the tyrosine-phosphorylated cytoplasmic domain of CEACAM3 in intact cells, but furthermore, FRET measurements allow the subcellular localization of this process during bacterial infection. FRET-based assays are valuable tools to resolve bacteria-induced protein-protein interactions in the context of the intact host cell.

## Background

Pathogenic bacteria tightly interact with their host, often exploiting adhesin-mediated engagement of eukaryotic surface receptors to trigger intracellular signaling events [1]. As bacteria-induced responses are of critical importance during the initiation and progression of the infection, signaling processes in the host cell are usually studied in molecular detail. Both biochemical and genetic approaches have shed light on protein-protein interactions and signaling connections that occur in infected eukaryotic cells. However, widely used biochemical approaches to investigate protein-protein interactions such as glutathione S-transferase (GST)-pull-down assays or coimmunoprecipitation from cell lysates have two major drawbacks: firstly, it is always possible that the two associated proteins are not directly interacting, but rather are linked by a third protein; secondly, biochemical approaches disrupt the cellular context and therefore lack spatial resolution. Similarly, genetic methods such as yeast two-hybrid screens, although applicable even in a high-throughput format, do not provide any information on where these processes occur under physiological conditions at the subcellular level.

By contrast, the introduction of green fluorescent protein (GFP) from *Aequorea victoria*, has greatly facilitated the microscopic investigation of proteins in living cells. Though the use of GFP and its spectral variants allows the observation of colocalization of multiple proteins in real time, the resolution of light microscopes (about 250 nm) is too low to prove a direct interaction of two colocalized putative binding partners. Therefore, there is a need for methods that combine the power of biochemical studies to pinpoint molecular interactions with the ability to study the subcellular context as provided by fluorescence microscopy.

Within the last few years, the phenomenon of fluorescence resonance energy transfer (FRET), first described by Förster in 1948, has garnered increasing interest as a method to address protein-protein interactions in the context of the cell [2-4]. During FRET, energy is transferred from a donor fluorophore in its excited state in a non-radiative way by dipole-dipole interactions to an acceptor molecule [5]. The efficiency of fluorescence resonance energy transfer is defined by: E = 1/[1 + (r/R_0_)^6^]. Apparently, the efficiency of energy transfer depends on the sixth power of the distance 'r' separating the donor and the acceptor molecule. Therefore, FRET only takes place to a significant extent if molecules are spaced within a few nm (about 1 to 10 nm) [6]. The additional parameter 'R_0_', called the Förster radius, is defined as the distance where efficiency of energy transfer from donor to acceptor is 50%. R_0 _is FRET-pair specific and is influenced by the spectral overlap of donor emission and acceptor excitation, the quantum yield of the donor, the absorption coefficient of the acceptor and the relative orientation of donor and acceptor. As a consequence, FRET is only likely to occur if two proteins labeled with a donor and an appropriate acceptor molecule are in direct contact.

To exploit this methodology in the study of pathogen-induced host cell signaling and to provide a general framework on how to approach FRET analysis in the context of receptor-initiated signaling cascades, we have used the example of carcinoembryonic antigen-related cell adhesion molecule (CEACAM)-mediated contact with the Gram-negative pathogen *Neisseria gonorrhoeae*. Over the last few years, our group and others have demonstrated that CEACAM3, a granulocyte-expressed member of this receptor family, functions as an opsonin-independent phagocytic receptor [7,8]. CEACAM3 recognizes colony opacity associated (Opa) proteins of *N. gonorrhoeae *(Ngo) as well as additional outer membrane adhesins of other Gram-negative bacteria and, upon binding of bacteria, initiates an intracellular signaling cascade [9].

Efficient uptake of CEACAM3-bound bacteria depends on an immunoreceptor tyrosine-based activation motif (ITAM)-like sequence in the cytoplasmic part of the receptor, which is phosphorylated within minutes of receptor engagement [7]. Biochemical analyses have demonstrated that Src homology 2 (SH2) domains of several signaling molecules, including the protein tyrosine kinases (PTKs) c-Src and Hck, are able to bind to the tyrosine-phosphorylated cytoplasmic domain of CEACAM3 [10]. As well as the SH2 domain of Src PTKs, the SH2 domains of phosphatidylinositol-3 kinase, phospholipase Cγ, and Syk have also been found to colocalize with the receptor upon bacterial binding [11,12]. However, it is unclear if this colocalization is indeed due to direct interaction of the respective signaling molecule with the phosphorylated receptor, or if there are additional molecules involved.

**Figure 1:**
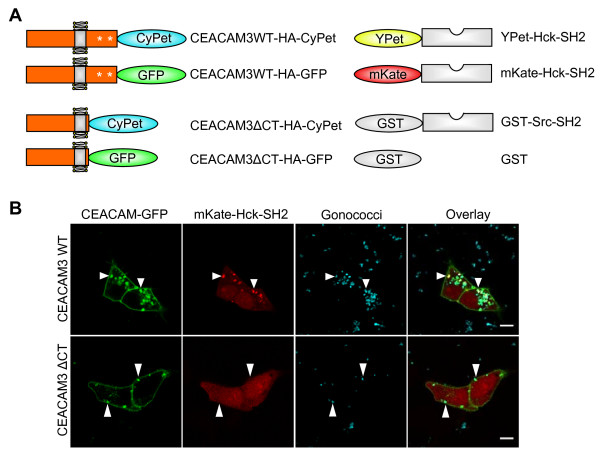
**Overview of the used constructs**. **(a) **Wild type carcinoembryonic antigen-related cell adhesion molecule 3 (CEACAM3 WT) comprising an immunoreceptor tyrosine-based activation motif (ITAM)-like sequence in its cytoplasmic domain (CT) or CEACAM3 ΔCT lacking the entire cytoplasmic domain were C-terminally tagged with cyan fluorescent protein (CyPet), as a fluorescence resonance energy transfer (FRET) donor, or enhanced green fluorescent protein (EGFP), respectively. Two specific tyrosine residues within the ITAM-like sequence are known to be phosphorylated upon receptor activation (indicated by asterisks). Hck-Src homology 2 (SH2) domain was cloned as a 3' fusion to far-red fluorescent protein (mKate) or enhanced yellow fluorescent protein (YPet), which served as FRET acceptor. **(b) **The SH2 domain of Hck is recruited to CEACAM3, when cells are infected with Opa_CEA_-expressing *Neisseria gonorrhoeae*. 293T cells were cotransfected with expression plasmids encoding the indicated GFP-tagged CEACAM3 variants and mKate-Hck-SH2 as indicated. Cells were infected with AF647-labeled Opa_CEA_-expressing *N. gonorrhoeae *at a multiplicity of infection of 30 bacteria/cell. Scale bar corresponds to 10 μm.

In the present investigation, we took advantage of a novel FRET pair of fluorophores, enhanced cyan fluorescent protein (CyPet) and enhanced yellow fluorescent protein (YPet), which have been recently developed by random mutagenesis and which display enhanced FRET efficiency compared to the often used cyan fluorescent protein (CFP)-yellow fluorescent protein (YFP) pair [13]. Tagging the cytoplasmic domain of CEACAM3 with CyPet and coexpressing a YPet-SH2 domain fusion protein (Figure 1a), we observed FRET in cell lysates and by flow cytometry in intact cells expressing phosphorylated CEACAM3-CyPet together with YPet-SH2 Hck, but not with YPet-SH2 SH2 domain-containing leukocyte protein of 76 kDa (SLP-76). Importantly, FRET measurements in infected cells using acceptor bleaching revealed the direct interaction between CEACAM3 and Hck exclusively at sites of bacterial contact with host cell. Together, these data highlight the use of FRET approaches to visualize cellular signaling in response to bacterial host cell contact and provide conclusive evidence for a direct interaction between CEACAM3 and Hck upon *N. gonorrhoea*e infection.

## Results and Discussion

### Bacterial engagement of CEACAM3 is accompanied by Hck-SH2 recruitment

Receptor engagement by bacterial pathogens is known to trigger intracellular signaling cascades in the infected eukaryotic cell. Our group and others have shown previously that Src kinases are critical for CEACAM3-mediated uptake of *N. gonorrhoeae *[10,14,15]. Biochemically, GST-pull-down assays have demonstrated the ability of the Src kinase Hck to interact with the phosphorylated cytoplasmic domain (CT) of CEACAM3. To demonstrate the recruitment of Hck to CEACAM3 upon bacterial binding and to investigate the kinetics of this process, 293T cells were cotransfected with the cDNA of Hck-SH2-far-red fluorescent protein (mKate) together with CEACAM3 WT-GFP or CEACAM3 ΔCT-GFP, respectively (Figure 1a). At 2 days later, the cells were infected with Opa_CEA_-expressing *N. gonorrhoeae *and imaged once per min for 2 h using confocal microscopy (see Additional file 1).

Representative images of live cells during the infection process are shown in Figure 1b. Whereas Hck-SH2 strongly colocalizes with CEACAM3 WT at sites of bacterial contact, the unligated receptor does not recruit the kinase SH2 domain. In cells expressing CEACAM3 ΔCT, a mutant form of the receptor that lacks the complete cytoplasmic domain and that is not phosphorylated upon bacterial infection, Hck-SH2 is distributed evenly in the cytoplasm, even though the bacteria bind to the extracellular domain (Figure 1b and Additional file 2). Clearly, Hck-SH2 recruitment to the bacteria-bound CEACAM3 WT is transient (see Additional file 1). Within 5 to 10 min, the SH2 domain disappears from cell-associated bacteria suggesting that the CEACAM3-initiated signaling complex is changing its composition during bacterial internalization. It is also interesting to note that cells expressing CEACAM3 WT seem to polarize with regard to bacterial uptake: Hck-SH2 is recruited to one side of the cell, where efficient receptor clustering and internalization takes place. If this represents direct binding of the Hck-SH2 domain to the phosphorylated cytoplasmic domain of CEACAM3 or if the recruitment of Hck-SH2 is due to some other phosphoprotein, which is found in the vicinity of the receptor, remains unresolved. However, the results clearly demonstrate that the Hck SH2 domain is effectively recruited to CEACAM3-enriched parts of the cell membrane upon receptor engagement by bacteria.

### Hck-SH2 binds to phosphorylated cytoplasmic domain of CEACAM3

**Figure 2:**
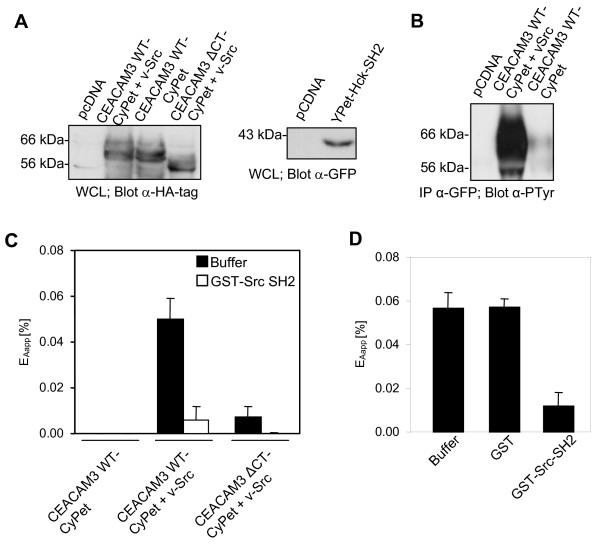
**Hck-Src homology 2 (SH2) domain interacts with the phosphorylated immunoreceptor tyrosine-based activation motif (ITAM)-like sequence of carcinoembryonic antigen-related cell adhesion molecule 3 (CEACAM3)**. 293T cells were cotransfected to express CEACAM3 wild type tagged with cyan fluorescent protein (CEACAM3 WT-CyPet) or CEACAM3 without the cytoplasmic domain (CEACAM3 ΔCT-CyPet), together with enhanced yellow fluorescent protein (YPet)-Hck-SH2 and v-Src as indicated. At 2 days after transfection cells were lysed. **(a) **Western blot of the whole cell lysates (WCL) with anti-hemagglutinin (HA) antibodies (upper panel) or anti-green fluorescent protein (GFP) antibodies (lower panel) confirmed expression of the indicated proteins after transfection. **(b) **CEACAM3 WT was immunoprecipitated from lysates and analyzed by western blotting with anti-phosphotyrosine antibodies. **(c) **Fluorescence of lysates harboring the indicated proteins was determined in three separate channels and apparent fluorescence resonance energy transfer (FRET) efficiency (E_Aapp_; black bars) was calculated as described. Purified glutathione S-transferase (GST)-Src-SH2 was added as a competitive inhibitor of YPet-Hck-SH2 to the lysates and measurements were repeated (open bars). **(d) **Purified GST, GST-Src-SH2, or buffer were added to lysates of cells expressing CEACAM3 WT-CyPet, YPet-Hck SH2 and v-Src. FRET efficiency (E_Aapp_) was calculated as in (c). Bars represent mean values ± standard error of the mean (SEM) of three independent experiments.

FRET is a powerful tool to investigate protein-protein interactions. However, the ability to observe FRET depends on multiple parameters, including the proper spatial orientation of the fluorescent molecules, which is hard to predict even for known interaction partners. In our case, the CyPet tag was added to the C-terminus of CEACAM3 that is in close proximity to the tyrosine residues of the ITAM-like sequence [9], whereas the YPet tag was added to the N-terminus of the SH2 domain of Hck (Figure 1a). To investigate, if the association of the Hck SH2 domain with the phosphorylated cytoplasmic domain of CEACAM3 can be monitored in the chosen configuration, we first used a simple *in vitro *FRET assay. Therefore, 293T cells were cotransfected with CEACAM3 WT-CyPet and YPet-Hck-SH2 together with or without v-Src. Indeed, coexpression of v-Src leads to constitutive tyrosine phosphorylation of CEACAM3 in the absence of bacterial infection (Figure 2b). As a further control, we coexpressed CEACAM3 ΔCT-CyPet together with YPet-Hck-SH2 in the presence of v-Src. Cells were lysed 2 days after transfection, when equivalent amounts of the receptor or the Hck SH2 domain were expressed in the different samples (Figure 2a). The resulting cell lysates were analyzed for FRET using a spectrofluorometer. Importantly, FRET signals were only detected in samples coexpressing CEACAM3 WT together with YPet-Hck-SH2 and v-Src (Figure 2c). By contrast, no FRET was detected in the absence of v-Src under conditions where CEACAM3 WT is not tyrosine phosphorylated. For CEACAM3 ΔCT, marginal FRET was detected both in the presence or absence of v-Src. These results suggest that efficient FRET between CyPet and YPet occurs only upon interaction of tyrosine-phosphorylated CEACAM3 WT with Hck-SH2, demonstrating that these two molecules are closely associated.

The method of measuring FRET between two potential interaction partners in cell lysates is less labor intensive than other biochemical approaches such as GST-pull-down assays or coimmunoprecipitations. Nevertheless, the sensitized emission generated by FRET in these samples cannot be observed directly. This is mostly due to the fact that the signal in the FRET channel is contaminated by spectral bleed through of donor emission and direct excitation of the acceptor at the excitation wavelength of the donor. Therefore, the signal has to be adjusted with specific correction factors derived from equivalent lysates containing either the donor or the acceptor only [16]. The sensitized emission calculated by linear unmixing of the signal in the FRET channel is further normalized to acceptor intensity to obtain FRET efficiency that can be compared between different samples [17].

To further assure that the calculated FRET efficiency between CEACAM3 WT-CyPet and YPet-Hck SH2 is due to an SH2 domain-mediated molecular interaction, we introduced an additional internal control. In this regard, we took advantage of a recombinant glutathione S-transferase fusion protein of the c-Src-SH2 (GST-Src SH2) that has shown strong binding to phosphorylated CEACAM3 WT in *in vitro *GST-pull-down assays [10]. We reasoned that an excess of non-fluorescent GST-Src SH2 added to the lysates should act as a specific competitive inhibitor displacing YPet-Hck-SH2 from the phosphorylated CEACAM3 WT. In line with this assumption, FRET between CEACAM3 WT-CyPet and YPet-Hck SH2 was almost completely abolished upon addition of the c-Src SH2 domain (Figure 2c). As a further control, similar amounts of GST or GST-Src SH2 were added to lysates of cells expressing CEACAM3 WT-CyPet, YPet-Hck SH2 and v-Src. Only in the case of GST-Src SH2 was a dramatic decrease of FRET efficiency observed, whereas GST alone had no effect on FRET (Figure 2d). These results demonstrate that FRET observed in whole cell lysates is due to a specific interaction between the YPet-tagged SH2 domain of Hck and the tyrosine-phosphorylated cytoplasmic domain of CEACAM3 WT-CyPet. Accordingly, we confirmed a tight binding between the SH2 domain of the Src family protein tyrosine kinase (PTK) Hck and CEACAM3.

### FRET between CEACAM3 and Hck-SH2 occurs in intact cells

**Figure 3:**
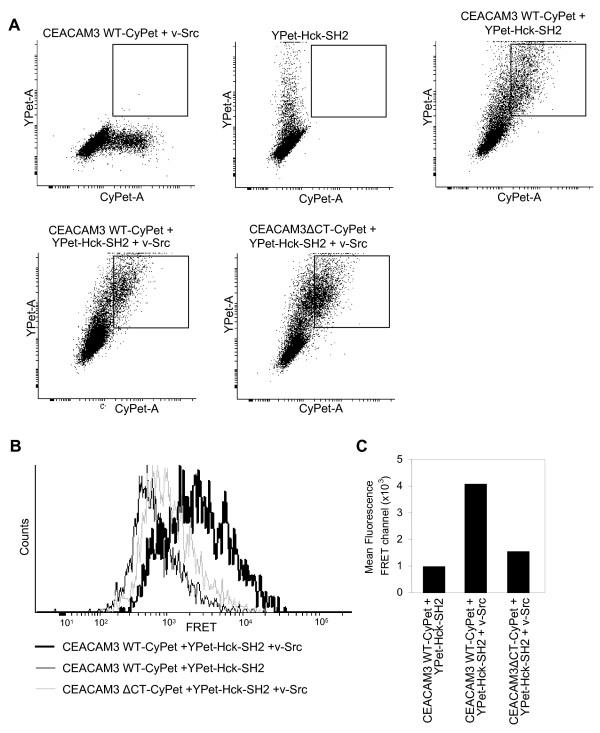
**Hck-Src homology 2 (SH2) domain interacts with the phosphorylated immunoreceptor tyrosine-based activation motif (ITAM)-like sequence of carcinoembryonic antigen-related cell adhesion molecule 3 (CEACAM3) in intact cells**. 293T cells were cotransfected to express CEACAM3 wild type tagged with cyan fluorescent protein (CEACAM3 WT-CyPet) or CEACAM3 without the cytoplasmic domain (CEACAM3 ΔCT-CyPet), together with enhanced yellow fluorescent protein (YPet)-Hck-SH2 and v-Src as indicated. At 2 days later, cells were analyzed by flow cytometry. **(a) **Cell populations expressing donor or acceptor constructs alone or cotransfected with CyPet-encoding and YPet-encoding constructs were identified. Dot plots show the CyPet and YPet fluorescence of the indicated samples. Squares indicate the population of CyPet and YPet double-positive cells coexpressing CEACAM3 WT and YPet-Hck-SH2. **(b) **Histogram of fluorescence intensity in the fluorescence resonance energy transfer (FRET) channel of gated CyPet and YPet double-positive cells as shown in (a). **(c) **Mean fluorescence intensity in the FRET channel of gated CyPet and YPet double-positive cells as shown in (a).

In cell lysates, the cellular context of membrane receptors and cytoplasmic signaling molecules is disrupted, potentially enabling interactions of protein partners that are not found in the same subcellular location. To investigate if the intimate association between CEACAM3 and Hck also occurs in intact cells, 293T cells were cotransfected as above and the intact cells were analyzed by flow cytometry (Figure 3a). Similar to the FRET determination in cell lysates, the intensity in the FRET channel is corrected for spectral bleedthrough and cross-excitation by using control cells expressing either fluorescent protein alone. Cells expressing both constructs were identified on the basis of CyPet and YPet fluorescence intensity (Figure 3a). In the presence or absence of v-Src, similar amounts of CyPet-positive and YPet-positive cells were observed and the double-positive cell populations in the samples did not differ with regard to the CyPet or YPet intensities measured (Figure 3a). Therefore, double-positive cells (expressing YPet-Hck-SH2 together with CEACAM3 WT-CyPet or CEACAM3 ΔCT-CyPet) were gated and their fluorescence intensity recorded in the FRET channel detecting YPet emission (525/50 nm) during excitation of CyPet (405 nm) (Figure 3b). Whereas only low fluorescence intensity was observed for cells transfected with YPet-Hck-SH2 and CEACAM3 WT-CyPet in the absence of v-Src, a strong increase in the FRET signal was obtained for cells coexpressing CEACAM3 WT-CyPet and YPet-Hck-SH2 together with v-Src (Figure 3b, c). Furthermore, cells expressing CEACAM3 ΔCT together with YPet-Hck-SH2 and v-Src showed no increase in fluorescence intensity in the FRET channel (Figure 3b). Though a fourfold increase in the mean fluorescence intensity in the FRET channel was observed in samples where CEACAM3 WT-CyPet, YPet Hck SH2, and v-Src are coexpressed, the extent of FRET might still be underestimated. This could be due to the fact that not every gated cell positive for the donor and acceptor fluorophore also expresses v-Src. Nevertheless, the flow cytometric analysis demonstrates intimate binding of Hck-SH2 to the phosphorylated cytoplasmic domain of CEACAM3 in intact cells. This suggests that recruitment of the Hck-SH2 domain to the sites of CEACAM3 engagement by bacteria as observed by live cell microscopy might be linked to the direct binding of these two proteins.

### FRET acceptor bleaching measurements reveal direct association between Hck-SH2 and CEACAM3 at sites of bacterial contact

**Figure 4:**
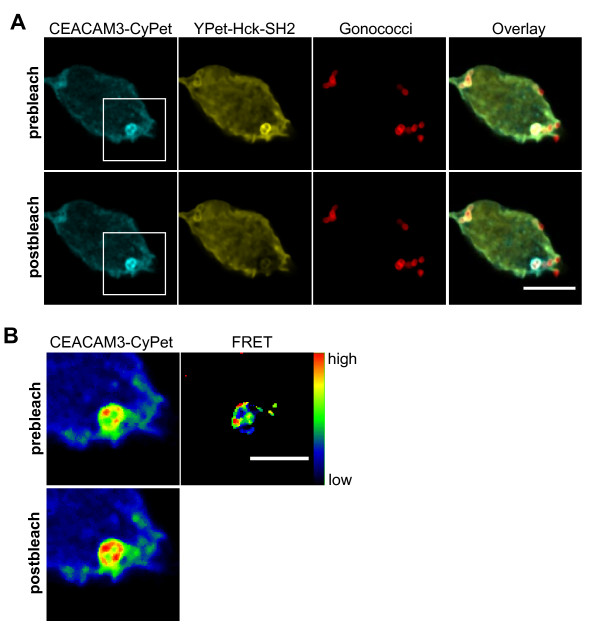
**The Hck Src homology 2 (SH2) domain directly associates with carcinoembryonic antigen-related cell adhesion molecule 3 (CEACAM3) at sites of bacterial contact**. **(a) **293T cells were cotransfected with expression plasmids for CEACAM3 wild type tagged with cyan fluorescent protein (WT-CyPet) and enhanced yellow fluorescent protein (YPet)-Hck-SH2. At 2 days later, cells were infected with AF647-labeled Opa_CEA_-expressing *Neisseria gonorrhoeae *for 30 min and fixed. The cell was imaged before and after photobleaching of the acceptor in a defined region, where bacteria were bound to CEACAM3. Scale bar: 10 μm. **(b) **Enlargement of the marked region in (a) in pseudocolor including the acceptor-bleached area. Fluorescence resonance energy transfer (FRET) was calculated as described in Methods. Scale bar corresponds to 5 μm.

The preceding experiments convincingly showed that FRET occurs between CEACAM3 WT-CyPet and YPet-Hck-SH2 upon constitutive receptor phosphorylation by v-Src. However, it was still unclear if and where this tight association takes place upon bacterial infection in intact cells. Therefore, NIH 3T3 cells were cotransfected with expression vectors for CEACAM3 WT-CyPet and YPet-Hck-SH2. At 2 days later, cells were infected with AlexaFluor647-NHS-labeled Opa_CEA_-expressing *N. gonorrhoeae *for 30 min and fixed. Images were recorded in all three fluorescence channels. In the region surrounding CEACAM3-bound bacteria, the acceptor fluorophore (YPet) was photochemically destroyed with a short high-intensity laser pulse (Figure 4a). If FRET occurs between donor and acceptor fluorophore, the destruction of the acceptor (YPet) should induce a significant increase in donor (CyPet) intensity. Importantly, such an increase in CyPet intensity upon laser bleaching of YPet can be observed exactly at the point, where bacteria were in contact with CEACAM3 (Figure 4b). These results not only verify an intimate binding of Hck-SH2 to CEACAM3 in response to receptor stimulation, but also localize this interaction precisely to the sites of bacteria-host cell contact.

FRET measurements based on sensitized emission have been recently used to detect phosphatidylinositol-3' kinase activation in response to bacterial infection [18]. The approach was based on the simultaneous recruitment of differentially labeled pleckstrin homology (PH) domains to the cell membrane upon generation of 3'-phosphoinositides. However, it was not designed to reveal protein-protein interactions. In our case, we employed a different methodology to microscopically detect FRET by acceptor photobleaching. In contrast to FRET determination based on sensitized emission, which requires extensive controls to exclude artifacts arising from variable concentrations and stoichiometry of acceptor or donor fluorophores in transiently transfected cells, acceptor bleaching displays FRET in a straightforward way. This is due to the fact that acceptor bleaching causes a positive signal due to an increase of donor fluorescence intensity following photochemical destruction of the acceptor. One reported caveat of acceptor photobleaching is the photoconversion of different YFP variants into a CFP-like species [19]. However, the photoconverted YFP with CFP-like properties appears to get excited primarily at 405 nm and to a lesser extent at 458 nm as used in the current investigation. In fact, we did not observe an increase of CyPet fluorescence in bleached regions without CEACAM3-bound bacteria (Figure 4b). Furthermore, we bleached cells expressing only the YPet acceptor construct and did not observe an increase of the signal in the CyPet channel upon excitation at 458 nm (data not shown). One clear advantage of acceptor bleaching is that it generates additional internal controls: first, donor intensity in regions with unbleached acceptor fluorophore should be unaffected; second, in bleached areas, where no protein-protein interactions take place, no alteration of donor fluorescence should be observed. Indeed, upon comparison of prebleaching and postbleaching pictures of the same region, an increase of donor intensity can only be observed where bacteria contact the host cell (Figure 4b). These results demonstrate that early host signaling in this situation is confined to the sites of tight pathogen binding to the receptor CEACAM3. It can be envisioned that such an approach would be also ideal to visualize and localize signaling events in response to other pathogenic bacteria. In particular receptor-dependent protein-protein interactions in the infected host cell that are induced upon binding of pathogens to host integrins [20-22], cadherins [23], tetraspanins [24], or proteoglycans [25] could yield valuable insight into the spatial organization of host responses. Furthermore, it would be highly desirable to study the subcellular distribution and place of action of bacterially secreted effector molecules [26,27]. This would require tagging of the secreted bacterial protein (for example, by tetracysteine motifs that could be selectively labeled by biarsenical dyes [28]) and would allow the validation of biochemically determined protein-protein interactions between bacterial effector molecules and host factors in the context of the intact infected cell.

As is often the case in signaling pathways, the recruitment of Hck-SH2 to CEACAM3 in living cells was transient (Additional file 1). Therefore, it would be of interest to analyze the kinetics of the Hck-SH2-CEACAM3 association by FRET in live infected cells. However, acceptor photobleaching destroys the acceptor fluorophore, preventing the continuous analysis of protein-protein interactions. Therefore, additional methodological approaches such as fluorescence lifetime imaging microscopy (FLIM) would be required. Measuring FRET according to the lifetime of the donor is independent of fluorophore concentrations and is non-destructive. However, the hardware requirements restrict the use of FLIM to dedicated microscope facilities that might be cautious of introducing live bacterial pathogens. This might limit the widespread use of FLIM in the realm of infection biology and makes the described approach of acceptor bleaching a valuable and feasible method to validate and subcellularly localize pathogen triggered signaling events in infected host cells.

## Conclusion

Elucidation of protein-protein interactions is of intense interest to understand signaling pathways in cells. Numerous biochemical and genetic approaches such as GST-pull-down assays, coimmunoprecipitation, yeast two-hybrid screens and protein microarrays are well established techniques for analyzing protein-protein interactions. However, utilization of FRET techniques not only allows the determination of intimate binding of two proteins, but also enables the verification of protein-protein interactions in the physiological context of the cell. Measuring FRET by acceptor bleaching allowed us to localize the biochemically established interaction between phosphorylated CEACAM3 and the Src PTK Hck to the sites of pathogen binding in infected host cells. Our results demonstrate that receptor-initiated signaling events are not only transient, as observed by the recruitment of the Hck SH2 domain in living cells, but the pathogen-induced protein-protein interaction is also spatially confined within the infected cell.

Therefore, this study refines our understanding of bacteria-induced signaling in eukaryotic cells and provides a rational framework to harness the potential of FRET in other close encounters between specialized microbes or their translocated effectors and the host organism.

## Methods

### Recombinant DNA

The coding sequences of CyPet and YPet were kindly provided by Patrick Daugherty (University of California, Santa Barbara, CA, USA). Upon polymerase chain reaction (PCR) amplification of CyPet (primers 5'-ACT**ACCGGT**CGTGGTGAGCAAGGGAGAG-3' and 5'-ACT**GCGGCCGC**TTATTTGTACAGTTCGTCC-3'), the coding sequence was inserted via *Age*I/*Not*I (restriction sites in primers are in bold) into pLPS-3' enhanced green fluorescent protein (EGFP) expression vector (Clontech, Mountain View, CA), thereby replacing the EGFP coding sequence and yielding pLPS-3'CyPet. YPet cDNA was PCR amplified (primers 5'-ACT**ACCGGT**ACCATGGTGAGCAAAG-3' and 5'-ATC**CTCGAG**ACTTATAGAGCTCGTTCATGC-3') and inserted in pEGFP-C1 loxP [29] via *Age*I/*Xho*I replacing EGFP and yielding pYPet loxP. Similarly, the cDNA of mKate (kindly provided by Dmitriy Chudakov, Shemyakin-Ovchinnikov Institute of Bioorganic Chemistry, Moscow, Russia) was amplified with PCR primers 5'-ATC**ACCGGT**ACCATGAGCGAGCTGATCAAG-3' and 5'-ACT**CTCGAG**TCTTGTGCCCCAGTTTGC-3' and inserted to obtain pmKate loxP.

The cDNA encoding the SH2 domain of human Hck was transferred by Cre-mediated recombination from pDNR-dual into pYPet loxP as described previously [10]. GST and the GST fusion protein of the SH2 domain of human c-Src, the v-Src and the CEACAM3-GFP expression constructs, as well as the hemagglutinin (HA)-tagged CEACAM3 variants in pBluescript were described previously [8,10,29]. CEACAM3 wild type (CEACAM3 WT) and CEACAM3 lacking the cytoplasmic domain (CEACAM3 ΔCT) were amplified with primers 5'-GAAGTTATCAGTCGATACCATGGGGCCCCCCTCAGCC-3' and 5'-ATGGTCTAGAAAGCTTGCAGCGTAATCTGGAACGTCATATGG-3' from the respective cDNA in pBluescript and subcloned in pDNR-Dual using the InFusion kit (Clontech). The cDNAs were subsequently transferred to pLPS-3'CyPet by Cre-mediated recombination to yield CEACAM3 WT-HA-CyPet or CEACAM3 ΔCT-HA-CyPet, respectively [21].

### Cell culture and transfection

The human embryonic kidney cell line 293T (293T cells) was grown in Dulbecco modified Eagle medium (DMEM)/10% calf serum (CS) at 37°C, 5% CO_2_. Cells were subcultured every 2 to 3 days. Transfection with expression vectors for CEACAM3, SH2 domains, v-Src or the empty control vector (pCDNA) was accomplished by standard calcium phosphate coprecipitation using a total amount of 6 μg plasmid/10 cm culture dish as previously described [8]. Cells were used 2 days after transfection. Expression was verified by western blotting as described previously.

The murine fibroblast cell line NIH 3T3 was grown in DMEM/10% fetal calf serum (FCS) supplemented with non-essential amino acids and sodium pyruvate on gelatine-coated culture dishes at 37°C, 5% CO_2_. Cells were subcultured every 2 to 3 days. NIH 3T3 cells were transfected with expression vectors using Metafectene Pro (Cambio, Cambridge, UK) according to the manufacturer's instructions.

### Bacteria

Opa_CEA_-expressing (Opa_52_), non-piliated *N. gonorrhoeae *MS11-B2.1 (strain N309) was obtained from T. F. Meyer (MPI Infektionsbiologie, Berlin, Germany). Bacteria were grown at 37°C, 5% CO_2 _on GC-Agar (Gibco BRL, Paisley, UK) supplemented with vitamins and appropriate antibiotics. For labeling, bacteria (2 × 10^8^/ml) were washed with sterile phosphate-buffered saline (PBS) and suspended in AlexaFluor647-NHS (Invitrogen, Karlsruhe, Germany) in PBS. Suspensions were incubated at 37°C for 30 min in the dark under constant shaking. Prior to use, bacteria were washed three times with PBS.

### FRET measurements in whole cell lysates

Transfected cells were washed (160 mM 3-(*N*-morpholino)propanesulfonic acid (MOPS), 1 mM ethyleneglycol tetra-acetic acid (EGTA), pH 7,4) and lysed for 10 min using the same buffer supplemented with 1% Triton X-100, 1 mM NaVO_3 _and complete protease inhibitors. A total of 100 μl of each lysate were transferred to a 96-well plate and the fluorescence in the following channels was recorded using a Varioskan Flash (Thermo Scientific, Waltham, MA): donor channel (excitation (Ex)/emission (Em): 435 nm/477 nm), acceptor channel (Ex/Em: 500 nm/530 nm), FRET channel (Ex/Em: 435 nm/530 nm). Raw data were processed by subtracting the background fluorescence signals obtained from lysates of untransfected cells. Signal in the FRET channel (DA) was corrected for spectral bleedthrough of the donor (α) and cross-excitation of the acceptor (β) with samples expressing donor or acceptor construct only. Afterwards sensitized emission was normalized to acceptor signal. In brief, FRET efficiency was calculated as follows: E_Aapp _= (DA-α·DD-β·AA)/AA where DD = signal donor channel and AA = signal acceptor channel.

### Flow cytometric FRET measurements

Transfected 293T cells were trypsin treated, suspensions were washed with ice-cold PBS and the cells resuspended in fluorescence-activated cell sorting (FACS) buffer (PBS, 1% FCS, 0.05% NaN_3_). Samples were kept on ice in the dark until measured. The cell population was gated by forward and sideward scatter, and 10^4 ^cells were analyzed using a LSR II flow cytometer (Becton Dickinson, Heidelberg, Germany). CyPet was excited at 405 nm and emission detected at 450/50 nm. YPet was excited at 488 nm and emission detected at 525/50 nm. Cells transfected with the empty vector (pCDNA) were used for background correction. Cells expressing donor or acceptor construct only were used to compensate the signal in the FRET channel (excitation: 405 nm, emission: 525/50 nm) for spectral bleedthrough and cross-excitation. Cotransfected cells were identified on the basis of CyPet and YPet fluorescence and the fluorescence intensity of double-positive cells was determined in the FRET channel.

### Confocal microscopy

For colocalization experiments, transfected 293T cells were seeded in 3.5 cm culture dishes with a coverslip bottom (MatTek, Ashland, MA, USA) at 1.5 × 10^5 ^cells/dish 1 day before infection. The culture dishes had been coated with a combination of human fibronectin (4 μg/ml) and poly-L-Lysine (10 μg/ml) in PBS at 37°C for 2 h. Cells were infected with AlexaFluor647-labeled *N. gonorrhoeae *and the infection process was monitored for 2 h with a TCS SP5 confocal laser scanning microscope (Leica, Wetzlar, Germany) using a 63 ×, 1.4 NA PLAPO oil immersion objective lens. Fluorescence signals of labeled specimens were serially recorded with appropriate excitation wavelengths and emission bands for EGFP, mKate and AlexaFluor647, respectively, to avoid bleedthrough. Images were processed with ImageJ (NIH, Bethesda, MD, USA).

For FRET acceptor bleaching studies, transfected NIH 3T3 cells were seeded on coated glass coverslips at 3 × 10^4 ^cells/well in 24-well plates 1 day before infection. Cells were infected with AlexaFluor647-labeled *N. gonorrhoeae *for 30 min and fixed with 4% paraformaldehyde in PBS. Following three washes, samples were embedded in mounting medium (Dako, Glostrup, Denmark). Acceptor bleaching was accomplished with a TCS SP5 using the implemented FRET acceptor bleaching wizard. Prebleach and postbleach images were serially recorded with excitation of CyPet at 458 nm and YPet at 514 nm with an argon laser and appropriate emission bands. Low laser intensities were used to avoid acquisition bleaching. The acceptor was bleached with high intensity at the 514 nm line. Cells expressing donor construct only were used to exclude donor bleaching under these conditions. Images were processed with ImageJ. To calculate FRET efficiency, donor prebleach (D_pre_) and postbleach (D_post_) images were smoothed by median filtering. Next, images were background subtracted and thresholded on fluorescence intensity. FRET efficiency (E) was calculated on a pixel-by-pixel basis as E = 1-(D_pre_/D_post_). Donor prebleach and postbleach images as well as FRET image are presented in pseudocolor for better visualization.

## Authors' contributions

AB and CRH conceived the study and designed the experiments, AB performed the experiments, TZ advised in microscopic FRET determination and evaluated the data, AB and CRH wrote the paper. All authors read and approved the final manuscript.

## Supplementary Material

Additional file 1 **Movie 1**. Hck-Src homology 2 (SH2) domain is transiently recruited to wild type carcinoembryonic antigen-related cell adhesion molecule 3 (CEACAM3 WT) at sites of bacterial infection. 293T cells were transfected to express far-red fluorescent protein (mKate)-Hck-SH2 and CEACAM3 WT-enhanced green fluorescent protein (EGFP). After addition of AF647-labeled Opa_CEA_-expressing *Neisseria gonorrhoeae *the infection process was monitored for 2 h. Scale bar: 10 μm. Click here for file

Additional file 2 **Movie 2**. Hck-Src homology 2 (SH2) domain is not recruited to carcinoembryonic antigen-related cell adhesion molecule 3 without the cytoplasmic domain (CEACAM3 ΔCT), even though bacteria still bind to the receptor. 293T cells were transfected to express far-red fluorescent protein (mKate)-Hck-SH2 and CEACAM3 ΔCT-enhanced green fluorescent protein (EGFP). After addition of AF647-labeled Opa_CEA_-expressing *Neisseria gonorrhoeae *the infection process was monitored for 2 h. Scale bar: 10 μm. Click here for file
